# Terminal Effector CD8 T Cells Defined by an IKZF2^+^IL-7R^−^ Transcriptional Signature Express FcγRIIIA, Expand in HIV Infection, and Mediate Potent HIV-Specific Antibody-Dependent Cellular Cytotoxicity

**DOI:** 10.4049/jimmunol.1900422

**Published:** 2019-09-13

**Authors:** Prossy Naluyima, Kerri G. Lal, Margaret C. Costanzo, Gustavo H. Kijak, Veronica D. Gonzalez, Kim Blom, Leigh Anne Eller, Matthew Creegan, Ting Hong, Dohoon Kim, Thomas C. Quinn, Niklas K. Björkström, Hans-Gustaf Ljunggren, David Serwadda, Elly T. Katabira, Nelson K. Sewankambo, Ronald H. Gray, Jared M. Baeten, Nelson L. Michael, Fred Wabwire-Mangen, Merlin L. Robb, Diane L. Bolton, Johan K. Sandberg, Michael A. Eller

**Affiliations:** * Makerere University Walter Reed Project, Kampala, Uganda;; † Center for Infectious Medicine, Department of Medicine, Karolinska Institutet, 17177 Stockholm, Sweden;; ‡ U.S. Military HIV Research Program, Walter Reed Army Institute of Research, Silver Spring, MD 20910;; § Henry M. Jackson Foundation for the Advancement of Military Medicine, Bethesda, MD 20817;; ¶ Department of Global Health, University of Washington School of Public Health, Seattle, WA 98195;; ‖ Laboratory of Immunoregulation, Division of Intramural Research, National Institute of Allergy and Infectious Diseases, National Institutes of Health, Bethesda, MD 20852;; # School of Medicine, Johns Hopkins University, Baltimore, MD 21205;; ** Rakai Health Sciences Program, Uganda Virus Research Institute, Entebbe, Uganda;; †† Faculty of Medicine, Makerere University College of Health Sciences, Kampala, Uganda;; ‡‡ Bloomberg School of Public Health, Johns Hopkins University, Baltimore, MD 21205;; §§ Department of Medicine, University of Washington School of Public Health, Seattle, WA 98195; and; ¶¶ Department of Epidemiology, University of Washington School of Public Health, Seattle, WA 98195

## Abstract

HIV-1 infection expands large populations of late-stage differentiated CD8 T cells that may persist long after viral escape from TCR recognition. In this study, we investigated whether such CD8 T cell populations can perform unconventional innate-like antiviral effector functions. Chronic untreated HIV-1 infection was associated with elevated numbers of CD45RA^+^CD57^+^ terminal effector CD8 T cells expressing FcγRIIIA (CD16). The FcγRIIIA^+^ CD8 T cells displayed a distinctive transcriptional profile between conventional CD8 T cells and NK cells, characterized by high levels of *IKZF2* and low expression of *IL7R*. This transcriptional profile translated into a distinct NKp80^+^ IL-7Rα^−^ surface phenotype with high expression of the Helios transcription factor. Interestingly, the FcγRIIIA^+^ CD8 T cells mediated HIV-specific Ab-dependent cellular cytotoxicity (ADCC) activity at levels comparable with NK cells on a per cell basis. The FcγRIIIA^+^ CD8 T cells were highly activated in a manner that correlated positively with expansion of the CD8 T cell compartment and with plasma levels of soluble mediators of antiviral immunity and inflammation such as IP-10, TNF, IL-6, and TNFRII. The frequency of FcγRIIIA^+^ CD8 T cells persisted as patients initiated suppressive antiretroviral therapy, although their activation levels declined. These data indicate that terminally differentiated effector CD8 T cells acquire enhanced innate cell-like characteristics during chronic viral infection and suggest that HIV-specific ADCC is a function CD8 T cells use to target HIV-infected cells. Furthermore, as the FcγRIIIA^+^ CD8 T cells persist in treatment, they contribute significantly to the ADCC-capable effector cell pool in patients on antiretroviral therapy.

## Key Points

- Chronic HIV-1 is associated with increased levels of FcγRIIIA^+^ CD8 T cells.
- FcγRIIIA^+^ CD8 T cells display an innate transcriptomic profile akin to NK cells.
- ADCC is mediated by FcγRIIIA^+^ CD8 T cells at levels comparable with NK cells.

## Introduction

The exquisite sensitivity and specificity of TCR-mediated sensing of infection is central to the function of T cells but can also, in some situations, limit their ability to provide effective immunity. This is evident in the context of HIV-1 infection, in which the appearance of HIV-1–specific T cells coincides with initial viral decline; however, the response fails to completely suppress or clear infection (1–4). Since the initial characterization of HIV-specific cytotoxic CD8 T cells in the late 1980s (5–9), the limitations in their ability to control viral replication and clear infection are evident (10, 11). High mutation rates in HIV-1 contribute to the ability of the virus to escape adaptive T cell responses (3, 12–14). Also, HIV-specific T cells become functionally impaired during chronic infection, additionally limiting their ability to control viral replication (15–17). Indeed, polyfunctional HIV-specific T cell responses are associated with better disease outcomes compared with those with a narrower functional breadth (18–20). In chronic HIV-1 infection, the replicating viral quasispecies have, to a large extent, mutated away from the originally transmitted viral sequence under T cell selection pressure, and this probably contributes to the accumulation of late-stage effector CD8 T cells with a skewed maturational phenotype (21, 22).

Persistent pathogen replication in chronic infections, such as untreated HIV-1 infection, engages T cell–mediated immune responses continuously with sustained antigenic challenge. Interestingly, some chronic infections have been associated with expansion of an unusual subset of CD8 T cells expressing CD16 (23–25). CD16 is the low-affinity IgG Fc receptor and exists in two isoforms, FcγRIIIA (CD16a) and FcγRIIIB (CD16b). CD16b is expressed exclusively by neutrophils and recognizes IgG-containing immune complexes, whereas CD16a is best characterized for its role in mediating Ab-dependent cellular cytotoxicity (ADCC) as a function of the innate immune system (26, 27, and reviewed in 28). NK cells are able to mediate a strong effector function in response to signaling through CD16-mediated stimulation. Whereas Fc receptors are generally not expressed by T cells, CD16 can sometimes be expressed by subsets of TCRαβ T cells (29–32). Growing evidence suggests the potential importance of ADCC in protection from HIV-1 infection (33, 34). Additionally, nonneutralizing Abs mediate an array of effector functions through their interactions with Fc receptors that may potentiate protection from HIV-1 infection or inhibit viral replication postinfection (35–40). Still, a better understanding of effector mechanisms, such as ADCC, involved in HIV-1 control is needed.

In this study, we hypothesized that late-stage differentiation of CD8 T cells may be associated with transcriptional changes that support innate-like effector functions in the T cell compartment. We demonstrate, in this study, that chronic, untreated HIV-1 infection is associated with the expansion of a late-stage differentiated CD8 T cell population expressing FcγRIIIA and that this population mediates HIV-specific ADCC. Furthermore, we show that the FcγRIIIA^+^ CD8 T cells display a hybrid CD8 T cell and NK cell transcriptional profile characterized by high expression of NKp80 and the transcription factor Helios.

## Materials and Methods

### Patients and samples

Study participants aged 15–49 y were enrolled in a prospective community-based cohort to assess the prevalence and incidence of HIV-1 infection in Rakai District, Uganda, from 1998 to 2004 (Table I) (41–43). Infected subjects were identified between 1997 and 2002 with continued annual follow up through 2008. Blood samples from 103 randomly selected HIV-1 seropositive individuals and 40 community-matched seronegative controls were obtained. PBMCs were then isolated and cryopreserved as described previously (44). None of the patients had received antiretroviral therapy (ART). HIV-1 testing was performed as described previously (43). Positive samples were subjected to the Amplicor HIV-1 Monitor test, version 1.5 (Roche Diagnostics, Indianapolis, IN). The HIV-1–infected study participants initiating ART were from the Couples Observation Study (COS) in Kampala Uganda as previously described (45). The index partner in each HIV-1–serodiscordant couple was followed up after the initiation of ART. Samples were collected; CD4 T cell counts determined and viral load assessments made at baseline, 6 and 12 mo after initiation of ART.

### Ethics statement

The study was approved by the following institutional review boards in the United States and Uganda: the institutional Review Boards of Uganda’s National Council for Science and Technology and the National AIDS Research Committee, as well as Division of Human Subjects Protection at the Walter Reed Army Institute of Research. All participants gave written-informed consent, or written-informed consent was obtained from the parent or legal guardian of those aged 17. For samples from the COS in Kampala, Uganda, all participants gave written-informed consent, and ethical approvals for the study were obtained from Uganda’s National Council for Science and Technology and the National AIDS Research Committee and the University of Washington.

### Flow cytometry and mAbs

Cryopreserved specimens were thawed and washed. Counts and viability were assessed on the Guava PCA (Guava Technologies, Hayward, CA), using Guava ViaCount reagent. Standard flow cytometry phenotyping was performed as previously described (46). Commercial mAbs (clone) used in flow cytometry were as follows: CCR5/CD195 BV421 (2D7), CCR7/CD197 FITC (150503), CD14 allophycocyanin H7 (MΦP9), CD14 Alexa Fluor 700 (M5E2), CD19 Alexa Fluor 700 (HIB19), CD16 allophycocyanin Cy7, PE-Cy5, Pacific Blue and BUV496 (3G8), CD161 PE-Cy5 (DX12), CD27 PerCP Cy5.5 (L128), PD-1/CD279 Alexa Fluor 647 and PE (EH12.1), CD3 AmCyan, allophycocyanin–H7, and PerCP-Cy5.5 (SK7), CD3 PE-CF594 (UCHT1), CD4 BV605 and allophycocyanin–H7 (SK3), CD38 allophycocyanin (HB7), CD45RA allophycocyanin (HI100), CD56 PE-Cy7 (NCAM16.2) and (B159), CD8 PE-Cy7 and PerCP-Cy5.5 (SK1), CD8 PE and PerCP-Cy5.5 (RPA-T8), CD8b PE (2ST8.5H7), HLA-DR FITC (G46-6), IL-7R/CD127 FITC and Alexa Fluor 647 (HIL-7R-M21), KIR2DL2/DS2/DL3 PE (DX27), NKG2D/CD314 PerCP Cy.5 (ID11), TCRαβ FITC and allophycocyanin (T10B9.1A-31), TRAIL/CD253 PE (RIK-2) (all from BD Biosciences, San Jose, CA); Aqua LIVE/DEAD viability stain, CD3 PE Texas Red (7D6), CD14 PE-Cy5 (Tuk4), and CD19 PE-Cy5 (SJ25-C1) were obtained from Invitrogen (Carlsbad, CA); CD4 ECD (SFCL12T4D11), NKG2A allophycocyanin (Z199), and NKp46/CD335 PE (BAB281) were all from Beckman Coulter (Brea, CA); CD27 Alexa Fluor 700 (O323), NKp80 PE (5D12), CD45RA BV785 (HI100), CD57 allophycocyanin, Pacific Blue and FITC (HCD57), CD8 allophycocyanin-H7 (SK1), CXCR3 FITC (G025H7), KIR3DL1 Alexa Fluor 700 (DX9), and T-bet FITC (4B10) were from BioLegend (San Diego, CA); eomesodermin (Eomes) PE (WD1928), Helios eFluor450 (22F6), KIR2DL1/DS1 PerCP Cy5.5 (HP-MA4), and perforin FITC (DG9) were from eBioscience (San Diego, CA). For assessment of transcription factors, cells were washed, permeabilized and fixed using an optimized kit (FOXP3 transcription factor staining buffer set) before intranuclear stain. Flow cytometry data were acquired with a BD LSR II instrument or a BD FACSCanto II instrument (BD Biosciences). Sorting was performed on a four-laser BD FACSAria II SORP (BD Biosciences) contained in a biosafety cabinet. Clinical lymphocyte immunophenotyping was performed using the FACS MultiSET System and run on a FACSCalibur using the single-platform Multitest four-color reagent in combination with Trucount tubes (BD Biosciences) (47).

### Soluble factor analysis

A custom multiplex cytokine array was used to quantify 16 analytes from cryopreserved plasma, including IFN-γ, IL-1α, IL-1β, IL-2, IL-4, IL-5, IL-6, IL-8, IL-10, IL-12p70, IL-15, IL-17, IP-10, MCP-1, TNF, and TNFRII, according to the manufacturer’s instructions (Quansys Biosciences, Logan, UT). Commercial single ELISAs were used to measure neopterin (GenWay Biotech, San Diego, CA), IFN-α, I-FABP, and sCD14 (R&D Systems). All samples were run in triplicate, and mean values were used for data analysis.

### Gene expression analysis

Targeted gene expression analysis was performed as previously described (48). Cells from seven donors were stained and four phenotypically distinct cell populations (CD8 T cells: CD45RA-CD57^−^, CD45RA^+^CD57^+^FcγRIIIA^−^, CD45RA^+^CD57^+^FcγRIIIA^+^, as well as CD56^dim^FcγRIIIA^+^ NK cells) (500–1000 cells per well) were sorted into wells containing 10 μl of reaction buffer (SuperScript III Reverse Transcriptase/Platinum Taq Mix, CellsDirect 2×Reaction Mix; Invitrogen). Reverse transcription and specific transcript amplification were performed using a thermocycler (GeneAmp PCR System 9700; Applied Biosystems) as follows: 50°C for 15 min, 95°C for 2 min, then 95°C for 15 s, 60°C for 30 s for 18 cycles. The amplified cDNA was loaded into Biomark 96.96 Dynamic Array chips using the NanoFlex IFC controller (Fluidigm). This microfluidic platform was then used to conduct quantitative PCR in nL reaction volumes. Threshold cycle, as a measurement of relative fluorescence intensity, was extracted from the Biomark real-time PCR analysis software. A panel of 96 preselected genes related to both NK cell and CD8 T cell biology was qualified as previously described, using a script provided courtesy of Mario Roederer (49). Subsequent data analysis was performed using JMP software (version 10). Initial analyses of the transcriptome data from the Fluidigm Biomark confirmed the quality of 74 of the 96 genes, although data on 22 genes were discarded because of lack of amplification.

### ADCC assays

Measurement of ADCC was performed using the PanToxiLux assay (OncoImmunin, Gaithersburg, MD) similar to the previously described assay (50). rHIV-1 BaL gp120 (catalog no. 4961; obtained through the National Institutes of Health AIDS Reagent Program, Division of AIDS, National Institute of Allergy and Infectious Diseases, National Institutes of Health) were used to coat target CEM.NKR_CCR5_ cells. Optimal concentration used to coat target cells was determined for each gp120 through an 11-point titration starting with 20 μg/ml and 2-fold serial dilution. After coating CEM.NKR_CCR5_ target cells with gp120 in 0.5% FBS–RPMI media, cells were labeled with TFL4 (OncoImmunin), a fluorescent target cell marker, for 15 min at 37°C and 5% CO_2_. Cells were then washed twice with 1× PBS and stained with the viability dye LIVE/DEAD Fixable Aqua Dead Cell Stain (Life Technologies) for 30 min at room temperature. After washing in 0.5% FBS–RPMI media, cells were counted as above, then resuspended to reach a final concentration of 8.0 × 10^5^ cells/ml. At this point, sorted effector cell populations (NK cells, CD45RA^+^CD57^+^ CD8 T cells, and CD45RA-CD57^−^ CD8 T cells) were washed in 0.5% FBS–RPMI media and resuspended to a final concentration of 24 × 10^6^ cells/ml for an E:T ratio of 30:1. In a 96-well polypropylene plate, 25 μl of both target and effector cell suspensions were both added to each well along with 75 μl of granzyme B substrate (OncoImmunin). After incubation for 5 min at room temperature, 25 μl of HIV-Ig (North American Biologicals, Miami, FL) at a 0.5 mg/ml dilution was added to each well, and the plate was incubated for another 15 min at room temperature. The plate was then spun at 300 × *g* for 1 min and placed at 37°C and 5% CO_2_ for 1 h. Cells were washed twice with wash buffer and acquired on the LSR II (BD Biosciences) on the same day. Fluorophores were detected using a 488-nm 50-mW laser with 515/20 filters to detect granzyme B substrate, a 406-nm 100-mW laser with 525/50 filters to detect Aqua LIVE/DEAD stain, and a 640-nm 40-mW laser with 670/30 filters to detect TFL4 stain. Because of the spectral properties of the fluorescent molecules used in this panel, manual compensation of detected signals was performed to analyze the data. Data were analyzed by using FlowJo 9.7.5 (Ashland, OR).

### Statistical analysis

Statistical analysis was performed using GraphPad Prism 6.0 (Version 6) for Macintosh (GraphPad Software, La Jolla, CA) or JMP software (version 10; SAS Institute, Cary, NC). Direct comparisons between two groups were performed using the nonparametric Mann–Whitney *U* test. Associations between groups were determined by Spearman rank correlation. To correct for multiple comparisons, the Benjamini–Hochberg false discovery rate (FDR) (51) was calculated for all observations. An FDR <0.05 was considered statistically significant. For paired observations, a paired *t* test was used. A *p* value <0.05 was considered statistically significant. Flow cytometry analysis and presentation of distributions were performed using SPICE version 5–1.2, downloaded from http://exon.niaid.nih.gov/spice (52). Comparison of distributions was performed using a Student *t* test and a partial permutation test as described previously (52).

## Results

### FcγRIIIA^+^ CD8 T cells expand in chronic untreated HIV-1 infection

**FIGURE 1.**
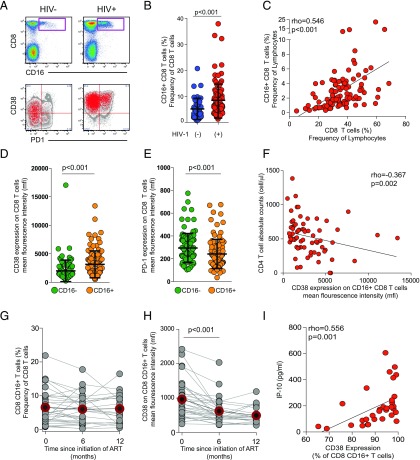
FcγRIIIA^+^ CD8 T cells expand numerically and persist in Ugandans with untreated HIV-1 infection. (**A**) Bivariate pseudocolor flow cytometry plots of FcγRIIIA^+^ CD8 T cells after gating on small lymphocytes that are Aqua LIVE/DEAD^−^TCR a/b^+^, CD8^+^CD3^+^ T cells in healthy donors (HIV^−^) (*n* = 40) and HIV-1–infected (HIV^+^) individuals (*n* = 103). Overlay plots of FcγRIIIA^+^ CD8 T cells (in red) and bulk CD8 T cells in gray for representative HIV^−^ and HIV^+^ donors. (**B**) Scatter plot of the frequency of FcγRIIIA^+^ CD8 T cells in HIV^+^ versus HIV^−^ healthy donors with lines at the mean and SD shown. (**C**) Correlation of the FcγRIIIA^+^ CD8 T cell subset frequency with the overall CD8 compartment frequency. (**D**) CD38 MFI and (**E**) PD-1 MFI in FcγRIIIA^+^ CD8 T cells (orange) as compared with the overall CD8 compartment (green) with lines at the mean and SD. (**F**) Correlation between FcγRIIIA^+^ CD8 T cells and absolute CD4 T cell counts. Longitudinal graph of the FcγRIIIA^+^ CD8 T cell subset frequency (**G**) and the CD38 MFI of FcγRIIIA^+^ CD8 T cell subset (**H**) in patients starting ART (*n* = 32) at baseline, 6, and 12 mo after ART initiation. Gray circles and lines represent individuals and red line and outlined, filled circle represents the median level. (**I**) Correlation between activation levels in FcγRIIIA^+^ CD8 T cells and TNFRII levels in plasma.

HIV-1 negative (*n* = 40) and HIV-1 positive (*n* = 103) individuals from a cohort in Rakai, Uganda, were chosen for the investigation of FcγRIIIA expression in CD8 T cells (Table I). The FcγRIIIA^+^ CD8 T cell population was identified as positive for CD3, TCRαβ, CD8, and FcγRIIIA and negative for CD14, CD19, and CD4 (Fig. 1A, Supplemental Fig. 1). FcγRIIIA expression was detectable in T cells from healthy donors at a median (range) frequency of 3.8% (0.7–20.7%) of CD8 T cells (Fig. 1B). Interestingly, this population was nearly doubled in HIV-1–infected donors, in which a median frequency of 5.9% (1.3–37.9%) of CD8 T cells expressed FcγRIIIA (*p* < 0.001) (Fig. 1B). This expansion was positively associated with the overall CD8 T cell expansion in HIV-1–infected patients (*p* < 0.001, rho = 0.546) (Fig. 1C). The HIV-1–associated expansion of FcγRIIIA^+^ CD8 T cells was not associated with the expression levels, measured as geometric mean fluorescence intensity (MFI), of FcγRIIIA on the surface of these cells (data not shown). There was no significant difference in FcγRIIIA expression levels (as measured by MFI) on FcγRIIIA^+^ CD8 T cells between HIV-1–infected and uninfected participants (data not shown). Interestingly, the FcγRIIIA^+^ CD8 T cells were more activated than their FcγRIIIA^−^ counterparts, as assessed by CD38 expression (*p* < 0.001) (Fig. 1D). They also expressed less of the inhibitory receptor PD-1 (*p* < 0.001) (Fig. 1E). The CD38 expression levels were inversely associated with CD4 counts, albeit weakly (*p* = 0.02, rho = −0.367), suggesting that the FcγRIIIA^+^ CD8 T cells become more activated as disease progresses (Fig. 1F).

**Table I.** Descriptive statistics for study population

	HIV-1­ Negative (*n* = 40)	HIV-1 Positive (*n* = 103)	HIV-1 Positive Initiating ART (*n* = 32)
Age (y), median (IQR)	30 (25–35)	31 (26–36)	32 (29–38)
Gender, no. (%)			
Female	20 (50)	65 (63)	14 (44)
Male	20 (50)	38 (37)	18 (56)
Viral load (log_10_/ml), median (IQR)*^a^*	NA	4.5 (4.1–5.12)	5.0 (4.1–5.3)
CD4 count (cells/μl), median (IQR)	NA	513 (375–670)	194 (139–240)

Whole blood from these participants was used to measure the expression of CD16 on CD8 T cells and characterize their activation profile.

^*a*^ Viral load was measured by Roche Amplicor Monitor version 1.5, limit of detection 400 copies/ml.

IQR, interquartile range; NA, not applicable.

To address the stability of the FcγRIIIA^+^ CD8 T cell pool over time, we studied a second cohort of Ugandan HIV-1–infected subjects (*n* = 32) located in Kampala, where longitudinal samples were available from before and after initiation of ART (Table I). These patients displayed a stable population of FcγRIIIA^+^ CD8 T cells over 12 mo of ART (Fig. 1G). However, of note, the activation levels of these FcγRIIIA^+^ CD8 T cells declined over the course of treatment, as measured by CD38 expression (*p* < 0.001) (Fig. 1H). These data show that HIV-1–infected Ugandans have an expanded population of activated TCRαβ CD8 T cells expressing FcγRIIIA and that this population is stable over 12 mo of ART.

Next, multiplexed assays and ELISA were used to quantify a suite of 20 soluble factors in plasma in relation to the size and activation level of the FcγRIIIA^+^ CD8 T cell population in HIV-1–infected individuals. Although none of the analytes measured showed a relationship to the percentage of CD8 T cells expressing FcγRIIIA, several markers were directly associated with the activation levels of FcγRIIIA^+^ CD8 T cells (i.e., cells coexpressing CD38) (Table II). Statistically significant correlations between the frequency of FcγRIIIA^+^ CD8 T cells expressing CD38 and plasma levels of the inflammatory cytokines IL-6 (*p* = 0.011, rho = 0.446, FDR = 0.040), IP-10 (*p* < 0.001, rho = 0.582, FDR = 0.009), MCP-1 (*p* = 0.016, rho = 0.424, FDR = 0.048), TNF (*p* = 0.008, rho = 0.459, FDR = 0.036), and TNFRII (*p* = 0.001, rho = 0.556, FDR = 0.009) were observed (Fig. 1I, Table II). Similar correlations were observed for the MFI of CD38 on FcγRIIIA^+^ CD8 T cells and IP-10 (*p* = 0.001, rho = 0.547, FDR = 0.009), MCP-1 (*p* = 0.009, rho = 0.456, FDR = 0.032), TNF (*p* = 0.009, rho = 0.458, FDR = 0.032), and TNFRII (*p* < 0.001, rho = 0.569, FDR = 0.009). Thus, expansion and activation of the FcγRIIIA^+^ CD8 T cells is associated with plasma markers of HIV-driven systemic immune activation. In contrast, neither soluble markers of an innate antiviral response, such as IFN-α, nor the common indices of microbial translocation sCD14 and IFABP were associated with the size of the FcγRIIIA^+^ CD8 T cell population or the extent of their activation.

**Table II.** Correlative analysis between plasma-derived soluble factors and CD16^+^ CD8^+^ T cells in HIV^+^ donors

	CD16^+^CD8^+^ T cell (%)			CD16^+^CD8^+^ CD38^+^ T Cell (%)			CD16^+^CD8^+^ CD38^+^ T Cell (CD38 mfi)		
Cytokine	rho	*p* Value	FDR	rho	*p* Value	FDR	rho	*p* Value	FDR
IFN-γ	−0.217	0.234	0.904	−0.082	0.655	0.737	0.003	0.986	0.986
IL-1a	−0.137	0.455	0.904	−0.228	0.210	0.344	−0.154	0.400	0.600
IL-1b	−0.079	0.669	0.904	0.015	0.935	0.935	0.062	0.735	0.882
IL-2	NA	NA	NA	NA	NA	NA	NA	NA	NA
IL-4	−0.213	0.242	0.904	0.139	0.448	0.620	0.201	0.271	0.443
IL-5	−0.077	0.673	0.904	0.118	0.522	0.671	0.045	0.807	0.908
IL-6	0.149	0.417	0.904	0.446	**0.011**	**0.040**	0.378	**0.033**	0.099
IL-8	0.067	0.714	0.904	0.032	0.864	0.915	0.018	0.921	0.975
IL-10	−0.095	0.604	0.904	0.505	**0.003**	**0.018**	0.465	**0.007**	**0.032**
IL-12p70	−0.034	0.854	0.904	0.338	0.059	0.133	0.290	0.108	0.216
IL-15	0.044	0.812	0.904	0.373	0.036	0.093	0.242	0.182	0.328
IL-17	NA	NA	NA	NA	NA	NA	NA	NA	NA
IP-10	−0.006	0.975	0.975	0.582	**0.001**	**0.009**	0.547	**0.001**	**0.009**
MCP-1	−0.055	0.765	0.904	0.424	**0.016**	**0.048**	0.456	**0.009**	**0.032**
TNF-α	−0.176	0.336	0.904	0.459	**0.008**	**0.036**	0.458	**0.009**	**0.032**
TNFR-II	−0.199	0.276	0.904	0.556	**0.001**	**0.009**	0.569	**0.001**	**0.009**
IFABP	0.041	0.823	0.904	−0.157	0.390	0.585	−0.136	0.457	0.633
sCD14	−0.077	0.674	0.904	0.307	0.087	0.157	0.312	0.082	0.200
IFN-α	−0.071	0.706	0.904	0.094	0.616	0.737	0.069	0.714	0.882
Neopterin	0.106	0.563	0.904	0.317	0.077	0.154	0.305	0.089	0.200

mfi, geometric MFI; NA, not applicable.

Bold indicates statistical significance, *p* < 0.05.

### FcγRIIIA^+^ CD8 T cells are late-stage effector cells and characterized by expression of Helios

**FIGURE 2.**
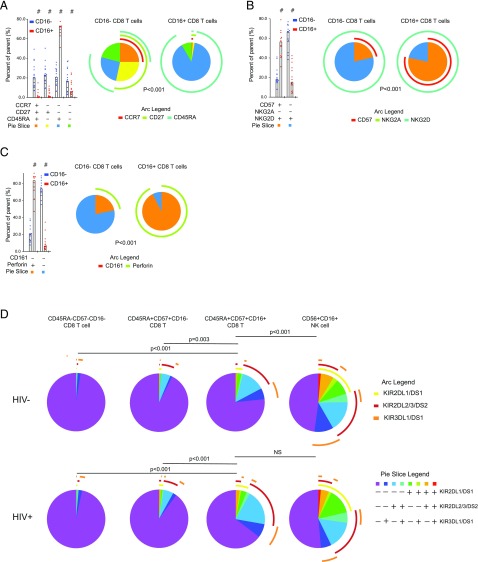
FcγRIIIA^+^ CD8 T cells display a late-stage effector phenotype in chronic untreated infection. A detailed phenotype of FcγRIIIA^+^ CD8 T cells after gating on small lymphocytes, singlets, Aqua LIVE/DEAD^−^, CD8^+^CD3^+^ T cells in HIV^+^ (*n* = 15) and HIV^−^ (*n* = 15) individuals was examined. (**A**) Expression of CD27, CCR7, and CD45RA in CD8 T cell subsets having or lacking FcγRIIIA surface expression. (**B**) Expression of CD57, NKG2A, and NKG2D in CD8 T cell subsets having or lacking FcγRIIIA surface expression. (**C**) Expression of CD161 and perforin in CD8 T cell subsets having or lacking FcγRIIIA surface expression. (**D**) Analysis of KIR surface expression patterns in CD45RA^+^CD57^+^FcγRIIIA^+^ CD8 T cells, CD45RA^+^CD57^+^FcγRIIIA^−^ CD8 T cells, CD56^dim^ NK cells, and CD45RA^−^CD57^−^ CD8 T cells.

Because of the significant expansion and activation of FcγRIIIA^+^ CD8 T cells in HIV-1–infected individuals, we next investigated the detailed phenotype of these cells in HIV-infected subjects from the Rakai cohort. The combinatorial coexpression pattern of CCR7, CD27, and CD45RA was significantly different between CD8 T cells positive or negative for FcγRIIIA (Fig. 2A, Supplemental Table I) (*p* < 0.001). Expression of CD45RA in the absence of CCR7 and CD27 was the dominant pattern among the FcγRIIIA^+^ CD8 T cells, consistent with a terminally differentiated status, whereas this phenotype was less common among CD8 T cells lacking FcγRIIIA (74% versus 18%, respectively) (*p* < 0.001). Next, the expression patterns of CD57, NKG2A, and NKG2D were evaluated, and the frequency of the subsets defined by these receptors were different in CD8 T cells expressing FcγRIIIA compared with those that did not (Fig. 2B) (*p* < 0.001). The majority of FcγRIIIA^+^ CD8 T cells expressed CD57 while maintaining NKG2D expression. In fact, all Boolean subsets containing CD57 expressing cells were higher in FcγRIIIA^+^ CD8 T cells compared with FcγRIIIA^−^ CD8 T cells (all *p* ≤ 0.001) (data not shown). The next panel examined CD161 and perforin, and comparison of the distribution of cell subsets expressing combinations of these two markers again revealed differences between the FcγRIIIA^+^ and FcγRIIIA^−^ CD8 T cells (Fig. 2C) (*p* < 0.001). The vast majority of FcγRIIIA^+^ CD8 T cells expressed perforin as compared with∼20% of FcγRIIIA^−^ CD8 T cells. In summary, FcγRIIIA^+^ CD8 T cells are distinct from their FcγRIIIA^−^ CD8 T cell counterparts by lack of CD27 expression, higher proportion of cells expressing CD57, and their predominantly perforin positivity.

The patterns of expression of maturation markers observed in FcγRIIIA^+^ CD8 T cells in HIV-1–infected donors were not significantly different from HIV-1–uninfected control subjects (all *p* values >0.05) (data not shown), suggesting that the elevated levels of FcγRIIIA^+^ CD8 T cells in infected individuals represent an expansion of a phenotypic cell subset retaining relatively normal characteristics. To address this question further, we investigated the expression of killer Ig-like receptors (KIRs) in CD8 T cells and NK cells expressing FcγRIIIA, as well as in late-stage differentiated CD8 T cells defined by coexpression of CD45RA and CD57 and memory CD8 T cells negative for these markers (Fig. 2D). In uninfected donors, T cell populations lacking FcγRIIIA had low levels of KIR expression, whereas NK cells had high KIR levels in diverse combinations. The FcγRIIIA^+^ CD8 T cells displayed a pattern intermediate between T cells and NK cells. Strikingly, this pattern was altered in HIV-1–infected subjects whose FcγRIIIA^+^ CD8 T cells had adopted a KIR coexpression profile very similar to that of NK cells (*p* < 0.001 for FcγRIIIA^+^ CD8 T cells in HIV-1–uninfected donors compared with HIV-1–infected donors; *p* = 0.250 for FcγRIIIA^+^ CD8 T cells compared with NK cells in HIV-1–infected donors).

**FIGURE 3.**
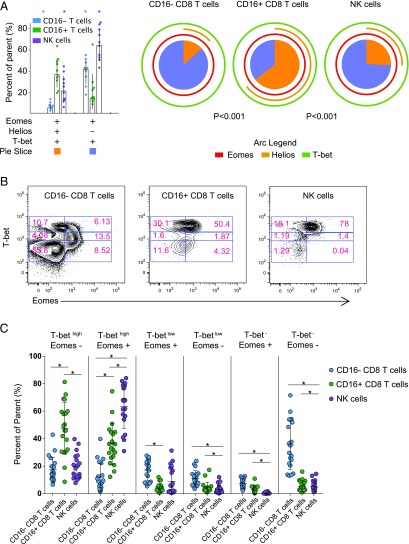
Transcription factors T-bet, Eomes, and Helios expression in CD8 T cells with or without FcγRIIIA and NK cells. In HIV^+^ (*n* = 10) and HIV^−^ (*n* = 10) individuals (**A**) expression of T-bet, Eomes, and Helios, assessed by intracellular staining, is presented for CD8 T cell subsets having or lacking FcγRIIIA (CD16) surface expression in comparison with NK cells. FcγRIIIA^−^ CD8 T cells (blue), FcγRIIIA^+^ CD8 T cells (green), and NK cells (purple) are displayed on a bar graph with individual points shown. Asterisks denote statistically significant differences of the FcγRIIIA^+^ CD8 T cells, with a *p* value <0.05). Major populations expressing all three transcription factors (orange box) and positive for T-bet and Eomes in the absence of Helios (lavender) are presented and correspond to slices of the pie chart. Individual expression of each transcription factor is shown by an arc (Eomes in red, Helios in gold, and T-bet in light green). A *p* value is presented for comparison of distribution of each part of the pie between groups. (**B**) Example flow cytometry plots showing the coordinated expression of T-bet and Eomes for FcγRIIIA^−^ CD8 T cells, FcγRIIIA^+^ CD8 T cells, and NK cells. (**C**) Scatter plot for FcγRIIIA^−^ CD8 T cells (blue), FcγRIIIA^+^ CD8 T cells (green), and NK cells (purple) for subsets of cells expressing high, medium, or low levels of T-bet and positive or negative for Eomes, with lines at the mean and SD shown. Line with * denotes statistical significance between cell populations. *p < 0.05.

T cell differentiation and maturation are controlled by a set of transcription factors, including T-bet, Eomes, and Helios. PBMC from HIV-infected donors were stained intracellularly for these transcription factors, and their expression patterns were analyzed in CD8 T cells lacking or expressing FcγRIIIA, as well as in NK cells (Fig. 3A). FcγRIIIA^+^ CD8 T cells displayed a T-bet, Eomes, and Helios expression pattern distinct from both the general CD8 T cell population and from CD56^dim^ NK cells, with higher levels of coexpression as compared with FcγRIIIA^−^ CD8 T cells. Coexpression of all three transcription factors was common in FcγRIIIA^+^ CD8 T cells and also relatively frequent in NK cells but uncommon in the general CD8 T cell pool. Notably, 61% of the FcγRIIIA^+^ CD8 T cells expressed Helios, and this was significantly higher compared with the FcγRIIIA^−^ CD8 T cells and NK cells (*p* < 0.001), in which a median of 10 and 28% expressed Helios, respectively. Characterization of T-bet and Eomes can be discriminated, based on a continuum of expression and varies on lymphocyte subsets (53). FcγRIIIA^+^ CD8 T cells were dominated by a high T-bet expression profile with variable Eomes expression that was very similar to CD16^+^ NK cells (Fig. 3B, 3C). HIV-1 infection status had minimal effect on T-bet and Eomes in these populations. FcγRIIIA^−^ CD8 T cells showed a much more variable expression pattern of both transcription factors, which may reflect the different states of maturation and differentiation within this compartment.

Altogether, these data indicate that the FcγRIIIA^+^ CD8 T cell population expanded in HIV-1–infected people is characterized by Helios expression and has a late-stage differentiated effector phenotype. This population mostly retains the characteristics seen in healthy donors as it expands during HIV-1 infection, although KIR expression is significantly elevated.

### The FcγRIIIA^+^ CD8 T cell transcriptome reveals a mixed effector CD8 T cell and NK cell character

**FIGURE 4.**
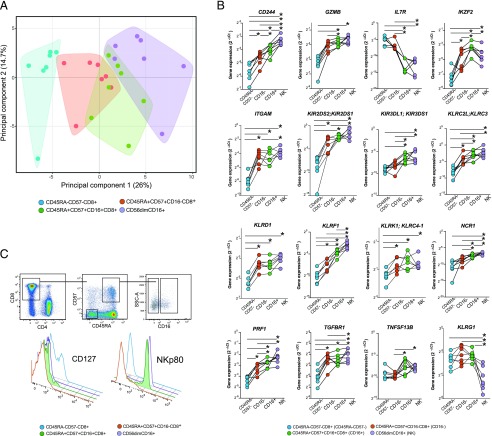
Transcriptome analysis reveals a mixed CD8 T cell and NK cell character in the FcγRIIIA^+^ CD8 T cells. Supervised expression analysis of 74 genes involved in the regulation and function of innate and adaptive immune responses in seven HIV-1–infected donors using the Fluidigm Biomark system. (**A**) PCA of the transcriptional data from four sorted cell populations reflecting CD45RA^−^CD57^−^ (blue), CD45RA^+^CD57^+^FcγRIIIA^−^ (red), CD45RA^+^CD57^+^FcγRIIIA^+^ (green), as well as CD56^dim^FcγRIIIA^+^ NK cells (purple). Polygons represent 95% confidence intervals in the data. (**B**) Expression of 10 selected genes in the same sorted subsets. (**C**) Successive flow cytometry gating strategy used for confirmation of IL-7Ra and KLRF1 genes at the protein level. Offset histograms showing the relative expression of IL-7Ra (CD127) and KLRF1 (NKp80) on CD8 T cells: CD45RA^−^CD57^−^ (blue), CD45RA^+^CD57^+^FcγRIIIA^−^ (red), CD45RA^+^CD57^+^FcγRIIIA^+^ (green/filled), as well as CD56^dim^FcγRIIIA^+^ NK cells (purple).

To better understand the identity of the FcγRIIIA^+^ CD8 T cells, we next analyzed their transcriptional profile by Fluidigm Biomark. A panel of 96 genes involved in T cell function or NK cell function was selected (Supplemental Table II), and the expression of these genes was analyzed in cell populations purified by flow cytometry sorting. For these analyses, cells from seven HIV-1–infected donors were sorted into four populations, 500–1000 cells per population: 1) CD45RA^+^CD57^+^ CD8 T cells expressing FcγRIIIA, 2) CD45RA^+^CD57^+^ CD8 T cells lacking expression of FcγRIIIA, 3) CD45RA-CD57^−^ CD8 T cells not expressing FcγRIIIA, and 4) CD56^dim^CD16^+^ NK cells. The data for 74 out of the 96 genes passed quality control, and principal component analysis (PCA) was performed on the total data set of expression of these 74 genes in all four of the cell subsets (Fig. 4A). Notably, the transcriptional profile of FcγRIIIA^+^ CD8 T cells overlapped with both the CD45RA^+^CD57^+^ CD8 T cells lacking expression of FcγRIIIA and the CD56^dim^CD16^+^ NK cells, whereas the CD45RA-CD57^−^ memory CD8 T cell subset was most distant. Principal component 1 contributed 26% of the variability in the data set, and component 2 contributed 14.7%. Expression of genes *GZMB*, *LAIR1*, *GZMK*, *PRF1*, and *CD244* contributed most to principal component 1, and genes *GZMK*, *IL-6ST*, *TGFB1*, *CD38*, and *CD160* contributed most to principal component 2.

A subset of genes showed expression patterns that segregated the FcγRIIIA^+^ CD8 T cell population from the NK cells and the FcγRIIIA^−^ CD8 T cell populations (Fig. 4B, Supplemental Fig. 2). Notably, the FcγRIIIA^+^ CD8 T cell displayed significantly higher *IKZF2* expression than any of the three other reference populations and lower *IL-7R* expression than the other T cell populations and at levels similar to CD56^dim^CD16^+^ NK cells. Regarding a range of genes encoding NK cell–associated receptors, including *KIR2DS2;KIR2DS1, KIR3DL1;KIR3DS1, KLRC2L;KLRC3*, *KLRD1, KLRF1, KLRK1;KLRC4-1*, and *NCR1*, the FcγRIIIA^+^ CD8 T cells showed a pattern intermediate between FcγRIIIA^−^ CD45RA^+^CD57^+^ CD8 T cells and the CD56^dim^CD16^+^ NK cells. In fact, *KLRF1* encoding the NK cell–associated receptor NKp80, expressed at the highest levels by CD56^dim^CD16^+^ NK cells, was expressed at significantly higher levels when compared with the FcγRIIIA^−^ terminal effector CD8 T cells and effector memory CD8 T cells. Compared with their FcγRIIIA^−^ counterparts, the FcγRIIIA^+^ CD8 T cells also expressed higher levels of genes involved in regulating T cell function, including *TNFSF13B.* Additionally, the FcγRIIIA^+^ CD8 T cells had significantly lower expression of *TGFBR1* than the CD56^dim^ NK cells, but levels were above that of the other CD8 T cells populations. Altogether, the gene expression analysis indicates that FcγRIIIA^+^ CD8 T cells have a transcriptional profile intermediate between effector CD8 T cells and CD56^dim^ NK cells.

Because of the distinct transcriptional signature of FcγRIIIA^+^ CD8 T cells, we were interested in confirming expression of the *IL-7R* and *KLRF1* genes at the protein level. We further examined 10 chronically HIV-1–infected and 10 uninfected individuals for surface expression of these receptors by flow cytometry. The majority of FcγRIIIA^+^ CD45RA^+^CD57^+^ CD8 T cells expressed NKp80 (median 68%) and lacked expression of the IL-7 receptor, CD127 (median 2%) (Fig. 4C). No differences were observed in FcγRIIIA^+^ CD45RA^+^CD57^+^ CD8 T cells expressing NKp80 or IL-7Rα between HIV-1 positive and negative individuals, and no relationship was observed between expression and markers of HIV-1 disease progression. IL-7Rα protein expression was similar between NK cells and CD45RA^+^CD57^+^ CD8 T cells, irrespective of FcγRIIIA^+^ expression. Interestingly, NKp80 was only found at appreciable levels in the T cells with the FcγRIIIA^+^ CD45RA^+^CD57^+^ phenotype. Together, the FcγRIIIA^+^ CD8 T cells have a distinct NKp80^+^ IL-7Rα^−^ character different from other effector CD8 T cells and more akin to CD56^dim^ NK cells.

### Potent HIV-specific ADCC activity mediated by FcγRIIIA^+^ CD8 T cells

**FIGURE 5.**
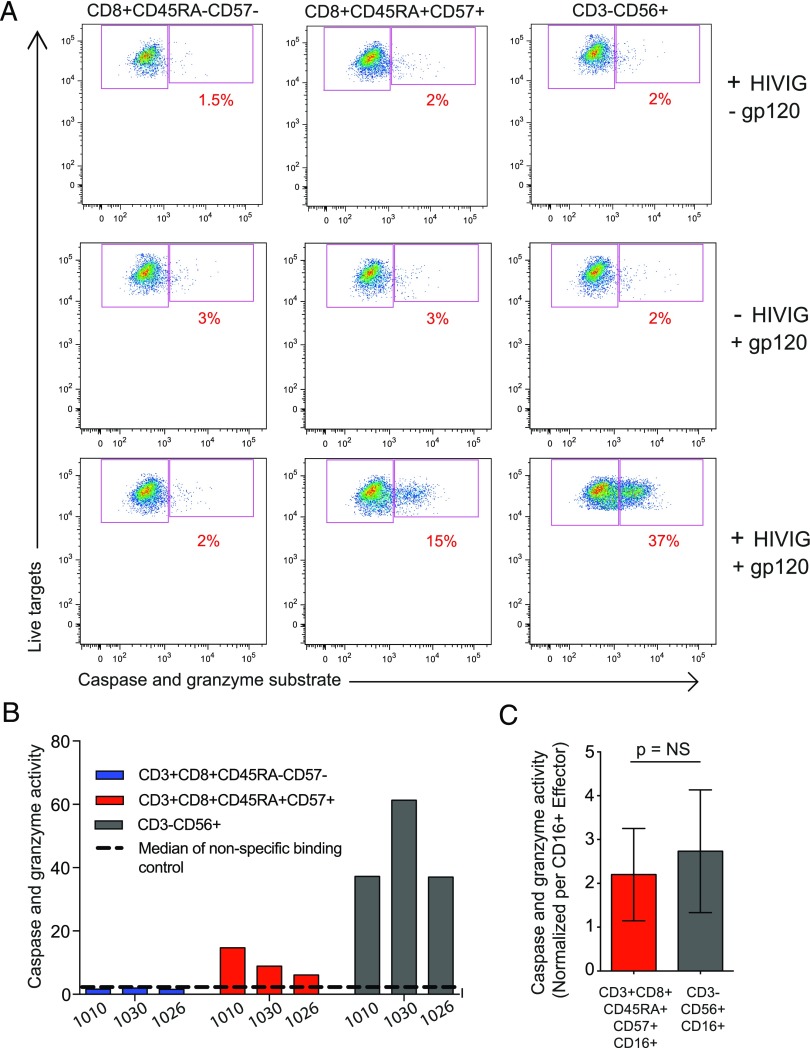
HIV-specific ADCC mediated by FcγRIIIA^+^ CD8 T cells. (**A**) Representative FACS plots of the cytolysis PanToxiLux assay from HIV^+^ (*n* = 3) individuals. (**B**) HIV-1 gp120-specific ADCC mediated by HIV-Ig. (**C**) Comparison of ADCC mediated by FcγRIIIA^+^ CD8 T cells and NK cells on a per FcγRIIIA^+^ cell basis.

ADCC is part of the repertoire of effector functions employed by NK cells to detect and target HIV-1–infected cells. Recent data indicating that nonneutralizing Ab-mediated effects may contribute to HIV vaccine efficacy have spurned a renewed interest in ADCC as a protective mechanism (54, 55). The present observation that HIV-1 infection drives the expansion of late-stage effector CD8 T cells with a hybrid NK cell–CD8 T cell character, including FcγRIIIA and lytic protein expression, suggests that CD8 T cells might actually mediate ADCC. To test this possibility, effector cell populations from HIV-1–infected donors were sorted by flow cytometry, and the ability of these cells to mediate ADCC against HIV BaL gp120-coated CEM.NKR_CCR5_ target cells was evaluated by the PanToxiLux granzyme B substrate cytotoxicity assay (Fig. 5A). To avoid FcγRIIIA downregulation or blocking because of staining, CD45RA^+^CD57^+^ CD8 T cells were sorted to enrich for FcγRIIIA^+^ cells (9–21% FcγRIIIA^+^) and then compared with FcγRIIIA^−^ CD45RA^−^CD57^−^ memory CD8 T cells and with NK cells sorted from the same donors. In the presence of HIV-Ig, the CD45RA^+^CD57^+^ cells from three HIV^+^ donors clearly mediated ADCC, as did the NK cells, whereas the CD45RA^−^CD57^−^ CD8 T cell population did not (Fig. 5A, 5B). As such, bulk CD45RA^+^CD57^+^ CD8 T cells performed ADCC lower than the NK cells (Fig. 5B). However, after adjusting for the frequency of FcγRIIIA expression in these populations, 9–21% in CD45RA^+^CD57^+^ CD8 T cells and 69–96% in CD56^dim^ NK cells, ADCC capacity of FcγRIIIA^+^ CD8 T cells was similar to that of FcγRIIIA^+^ NK cells (Fig. 5C). Interestingly, the FcγRIIIA^+^ MFI on FcγRIIIA^+^ CD8 T cells was significantly lower compared with FcγRIIIA^+^ MFI on CD56dim NK cells (*p* < 0.001). FcγRIIIA^+^ CD8 T cell ability to mediate ADCC, based on normalized FcγRIIIA^+^ MFI or the integrated MFI (frequency multiplied by the MFI), was as good as NK cells (data not shown). These data demonstrate that the FcγRIIIA^+^ CD8 T cell population expanding during chronic HIV-1 infection can mediate HIV-specific ADCC at levels comparable to NK cells.

## Discussion

CD8 T cells use a range of effector functions to combat viral infections, including cytolysis and effects mediated by cytokines and chemokines. A hallmark of these antiviral functions is that they depend on the exquisite Ag specificity of TCRs and their recognition of viral Ag in an MHC-restricted manner. In this study, we demonstrate that late-stage effector CD8 T cells acquire FcγRIIIA expression in HIV-1–infected individuals and use this Fc receptor to mediate HIV-specific ADCC in the absence of TCR recognition of Ag. Using a commercial in vitro assay, commonly used in assessing HIV-1 ADCC activity (50), we measured the effector capacity, on a per cell basis, of FcγRIIIA^+^ CD8 T cells to mediate Ag-specific ADCC against gp120-coated targets as efficiently as NK cells from the same donors. These findings indicate that in the context of chronic uncontrolled HIV-1 infection, a significant subset of CD8 T cells acquires innate characteristics and performs a function in the immune system normally associated with NK cells. Functional diversification of adaptive CD8 T cells may be important as therapeutic strategies evolve to include Ab-mediated mechanisms to eliminate HIV-1 reservoirs (56–58).

In the Ugandan population studied in this work, expression of FcγRIIIA occurs on∼5% of CD8 T cells from healthy donors, and this frequency is doubled in patients with chronic untreated HIV-1 infection. In fact, some patients have more than 30% of their CD8 T cells expressing FcγRIIIA. The finding that the size of this population is positively associated with the global CD8 T cell expansion in these patients suggests that the FcγRIIIA^+^ CD8 T cells expand in response to the chronic uncontrolled viral replication. These expanded cell populations have a terminally differentiated phenotype with frequent expression of CD45RA, CD57, and perforin but little expression of CD27 and CCR7, further supporting this notion. The phenotypic profile of these cells is similar between HIV-1–infected patients and healthy donors [data not shown and (23)]. However, we found one exception to this observation; the FcγRIIIA^+^ CD8 T cells adopt a KIR expression profile similar to NK cells in HIV-1–infected subjects, an observation not seen in healthy donors (Fig. 2D). The conditions in vivo during HIV-1 infection thus seem to drive not only an expansion of these cells but also expression of surface receptors beyond FcγRIIIA normally associated with NK cells and reflective of the rise in terminally differentiated CD8 T cells in chronic viral infections (59).

These functional and, to some extent phenotypic, similarities with NK cells led us to ask how the FcγRIIIA^+^ CD8 T cells relate to FcγRIIIA^−^ T cell subsets as well as FcγRIIIA^+^ NK cells on the transcriptional level. Based on a supervised transcriptional analysis of 74 genes in seven donors, the FcγRIIIA^+^ CD8 T cells appear to have a transcriptional program intermediate between late-stage effector CD8 T cells lacking FcγRIIIA and CD56^dim^ NK cell expressing FcγRIIIA. Most interestingly, transcript and protein levels for *KLRF1*, encoding the activating NKp80 receptor, were expressed at high levels similar to NK cells compared with effector memory or FcγRIIIA^−^ CD8 T cells. NKp80 has recently been shown to associate with the development and maturation of fully functional NK cells (60). Whereas FcγRIIIA^+^ CD8 T cells show some features similar to CD56^dim^ NK cells, PCA revealed that FcγRIIIA^+^ CD8 T cells, FcγRIIIA^−^ CD8 T cells, and CD56^dim^ NK cells were distinct from the CD45RA^−^CD57^−^ memory CD8 T cell population. Consistent with this notion, when the genes differentially expressed between the FcγRIIIA^+^ CD8 T cell and the effector memory T cell population were entered into the Reactome pathway analysis database, the *DAP12* pathway, implicated in activation of NK cells, was indicated as enriched in the FcγRIIIA^+^ CD8 T cells (Supplemental Fig. 2, Supplemental Table II) (61, 62). Furthermore, we observed the upregulation of 10 genes in FcγRIIIA^+^ CD8 T cells compared with the effector memory CD8 T cell population that are associated with NK-like rapid effector function and the “innateness gradient” defined by Gutierrez-Arcelus et al. (63) including *GZMB*, *PRF1*, *KIR3DL1*, *KLRK1*, *KLRD1*, *KLRF1*, *NCR1, KLRC2L;KLRC3*, *KIR2DS2*, and *ITGAM* (Supplemental Fig. 2, Supplemental Table II). Although the overall pattern is that FcγRIIIA^+^ CD8 T cells overlap with both FcγRIIIA^−^ T cells and CD56^dim^ NK cells, these cells also manifest distinctive features somewhere between innate and adaptive immune cells (64). A pattern that stands out is the high expression by FcγRIIIA^+^ CD8 T cells of the transcription factor Helios, encoded by the *IKZF2* gene, both at the protein and gene levels. These cells also have very low expression of IL-7Rα. The low IL-7Rα expression level is consistent with a model in which these cells are either maintained by non–IL-7–dependent factors or, rather, short-lived in vivo. Our finding that patients initiating ART largely maintain the expanded FcγRIIIA^+^ CD8 T cell population over 12 months suggests that these cells are not intrinsically short-lived, and thus may even be maintained by IL-7–independent mechanisms. This interpretation is supported by the recent finding of expansion of long-lived effector CD45RA^+^ CD8 T cells that are IL-7R^lo^ KLRG1^high^ in latent CMV and EBV infection, a population which phenotypically overlaps with the FcγRIIIA^+^ CD8 T cell identified in this study (65).

The expansion of FcγRIIIA^+^ CD8 T cells we observe in this study is reminiscent of the expansion of CD8 T cells with a similar phenotype in hepatitis C virus (HCV)–infected patients (23). Whereas HIV-1 and HCV differ in target cell tropism and mechanisms of pathogenesis, for example, they have in common establishment of chronic infections that are very difficult for the immune system to control. This is partly because of the shared features of rapid viral replication and high mutation rates. These features lead to selection of epitope immune escape variants that allow these viruses to avoid efficient recognition by clonally expanded populations of T cells. Viral quasispecies mutate away from the originally transmitted viral sequence under T cell selection pressure and some of the early responding epitope-specific T cell populations may thus lose their efficiency in targeting infected cells. Future studies are warranted to test the hypothesis that accumulation of FcγRIIIA^+^ CD8 T cells may be a clonally driven process and this could be addressed by TCR repertoire analysis. The FcγRIIIA^+^ CD8 T cells have a phenotype that would be expected from a T cell population expanded by Ag recognition, because they are largely negative for CD27 and CCR7, but positive for CD57, perforin and CD45RA. In the yellow fever virus vaccine model, the yellow fever vaccine–specific CD8 T cells are CD45RO^+^ during the peak of the effector response and then revert back to CD45RA expression as the Ag is cleared and memory is established (66, 67). This is consistent with a model in which CD45RA may be re-expressed when the T cells have not seen their cognate epitope for some time. This allows for the possibility that the FcγRIIIA^+^ CD8 T cells that expand numerically after HIV-1 infection as well as in HCV infection may be driven by viral epitopes that later accumulate escape mutations. Interestingly, the expanded FcγRIIIA^+^ CD8 T cell population described in this study displays frequent expression of inhibitory KIRs. Recent findings indicate that inhibitory KIR expression on CD8 T cells may enhance T cell survival in chronic viral infections and may facilitate the rescuing of an activated immunodominant T cell population after chronic Ag exposure (59). This population may then be viewed as a way for the immune system to repurpose Ag experienced T cells in defense against chronic viral infection (65). A recent study by Phaahla et al. (68) confirms the expansion of FcγRIIIA-expressing, ADCC-mediating CD8 T cells in an HIV positive South African cohort. Within the same cohort, FcγRIIIA expression declined on NK cells during HIV infection, which could potentially contribute to the observed decline of their capacity to mediate ADCC. These findings further support a model in which cytotoxic CD8 T cells are repurposed toward this innate-like function in the context of chronic HIV infection.

In summary, we describe a subset of late-stage differentiated CD8 T cells that acquire a distinctive hybrid NK cell and effector CD8 T cell character during untreated chronic HIV-1 infection, with expression of FcγRIIIA and potent HIV-specific ADCC activity. The development of this NK-like functionality in CD8 T cells may represent a way for the immune system to take full advantage of the cytolytic effector program of terminally differentiated cytolytic effector CD8 T cells during chronic viral infections and situations of epitope escape. In addition, the fact that expanded FcγRIIIA^+^ CD8 T cell populations persist after initiation of suppressive ART suggests that they may be engaged and contribute to Ab-based HIV cure strategies.

## Supplementary Material

Data Supplement
