# Supplementary material for: Terminal Effector CD8 T Cells Defined by an IKZF2+IL-7R− Transcriptional Signature Express FcγRIIIA, Expand in HIV Infection, and Mediate Potent HIV-Specific Antibody-Dependent Cellular Cytotoxicity
Source: J Immunol. 2019 Sep 13;203(8):2210–21. doi: 10.4049/jimmunol.1900422 (PMC6778306; doi:10.4049/jimmunol.1900422)
Supplement: Data Supplement [file JI_1900422.zip › JI_1900422_Supplemental_Material_1.pdf]

Supplemental Material

Supplemental Table 1. Boolean subset comparison across groups (Wilcoxon Rank Test Result)

1. HIV+ CCR7, CD27, and CD45RA expression

Category			P-value CD8 CD16+
CCR7	CD27	CD45RA	vs. CD16-
+	+	+	**0.000**
+	+	-	**0.000**
+	-	+	**0.000**
+	-	-	**0.013**
-	+	+	**0.001**
-	+	-	**0.000**
-	-	+	**0.000**
-	-	-	**0.038**

2. HIV+ CD57, NKG2A, and NKG2D expression

Category			P-value CD8 CD16+
CD57	NKG2A	NKG2D	vs. CD16-
+	+	+	**0.000**
+	+	-	**0.001**
+	-	+	**0.000**
+	-	-	**0.000**
-	+	+	0.281
-	+	-	0.130
-	-	+	**0.000**
-	-	-	**0.000**

3.HIV+ Perforin and CD161 expression

Category		P-value CD8 CD16+ vs. CD16-
NKG2A	NKG2D	
+	+	**0.000**
+	-	**0.000**
-	+	**0.000**
-	-	**0.000**

4. HIV+ Transcription Factors (Eomesodermin, Helios, and T-

Category			P-value CD8 CD16+	FDR of CD8 CD16+	P-value CD8 CD16+	FDR of CD8 CD16+
Eomes	Helios	T-bet	vs. CD16-	vs. CD16-	vs. NK cell	vs. NK cell
+	+	+	**0.000**	**0.001**	**0.019**	**0.0338**
+	+	-	**0.028**	**0.045**	**0.000**	**0.0015**
+	-	+	**0.007**	**0.014**	**0.001**	**0.0023**
+	-	-	0.112	0.138	**0.000**	**0.0015**
-	+	+	**0.001**	**0.002**	**0.000**	**0.0015**
-	+	-	0.880	0.880	0.496	0.5669
-	-	+	0.059	0.079	**0.049**	0.0713
-	-	-	**0.001**	**0.002**	0.597	0.6368

5. HIV+ Killer Immunoglobulin receptor (KIR) Expression

Category			P-value CD8+CD16+	FDR of CD8+CD16+	P-value CD8+CD16+	FDR of CD8+CD16+	P-value CD8+CD16+	FDR of CD8+CD16+
2DL1/DS1	2DL2/3/DS2	KIR3DL1	vs. CD8+CD16-	vs. CD8+CD16-	vs. CD8+CD45-	vs. CD8+CD45-	vs. NK cell	vs. NK cell
+	+	+	0.208	0.357	0.462	0.504	**0.001**	**0.005**
+	+	-	0.793	0.827	0.248	0.372	**0.016**	**0.038**
+	-	+	0.916	0.916	0.401	0.481	**0.001**	**0.005**
+	-	-	0.462	0.504	0.401	0.481	**0.027**	0.059
-	+	+	0.248	0.372	0.141	0.260	**0.012**	**0.032**
-	+	-	0.093	0.186	**0.001**	**0.005**	0.294	0.392
-	-	+	**0.003**	**0.012**	**0.012**	**0.032**	0.294	0.392
-	-	-	**0.005**	**0.017**	**0.001**	**0.005**	**0.001**	**0.005**

6. CD8+CD16+ Killer Immunoglobulin receptor (KIR) Expressi

Category			P-value HIV- vs.
2DL1/DS1	2DL2/3/DS2	KIR3DL1	HIV+
+	+	+	0.058
+	+	-	0.142
+	-	+	0.270
+	-	-	0.426
-	+	+	0.298
-	+	-	0.178
-	-	+	0.951
-	-	-	**0.006**

Supplemental Table 2. Gene expression RT-PCR reagents

Gene symbol	Gene Name	Entrez Gene ID	Assay ID
CD226	CD226 molecule	10666	Hs00170832_m1
EOMES	eomesodermin	8320	Hs00172872_m1
NCR1	natural cytotoxicity triggering receptor 1	9437	Hs00183118_m1
IKZF2	IKAROS family zinc finger 2 (Helios)	22807	Hs00212361_m1
KLRF1	killer cell lectin-like receptor subfamily F, member 1	51348	Hs00212979_m1
LAIR1	leukocyte-associated immunoglobulin-like receptor 1	3903	Hs00253790_m1
ITGAM	integrin, alpha M (complement component 3 receptor 3 subunit)	3684	Hs00355885_m1
KIR2DL4;LOC100287534	killer cell immunoglobulin-like receptor, two domains, long cytoplasmic tail, 4	3805	Hs00427106_m1
	killer cell immunoglobulin-like receptor 2DL4-like	100287534	
KIR3DL2	killer cell immunoglobulin-like receptor, three domains, long cytoplasmic tail, 2	3812	Hs00601497_gH
KIR3DX1	killer cell immunoglobulin-like receptor, three domains, X1	90011	Hs00736082_g1
KIR3DL1;KIR3DS1	killer cell immunoglobulin-like receptor, three domains, long cytoplasmic tail, 1	3811	Hs00744448_s1
	killer cell immunoglobulin-like receptor, three domains, short cytoplasmic tail, 1	3813	
IL7R	interleukin 7 receptor	3575	Hs00902334_m1
KLRG1	killer cell lectin-like receptor subfamily G, member 1	10219	Hs00929964_m1
IL2RG	interleukin 2 receptor, gamma	3561	Hs00953624_m1
IKZF1	IKAROS family zinc finger 1 (Ikaros)	10320	Hs00958474_m1
IL18R1	interleukin 18 receptor 1	8809	Hs00977691_m1
IL18RAP	interleukin 18 receptor accessory protein	8807	Hs00977695_m1
GZMA	granzyme A (granzyme 1, cytotoxic T-lymphocyte-associated serine esterase 3)	3001	Hs00989184_m1
IFNG	interferon, gamma	3458	Hs00989291_m1
TLR6	toll-like receptor 6	10333	Hs01039989_s1
LAIR2	leukocyte-associated immunoglobulin-like receptor 2	3904	Hs01102514_m1
LILRB1	leukocyte immunoglobulin-like receptor, subfamily B (with TM and ITIM domains), member 1	10859	Hs01848117_s1
KIR2DL3;KIR2DL2;KIR2DS2	killer cell immunoglobulin-like receptor, two domains, long cytoplasmic tail, 3	3804	Hs03407415_gH
	killer cell immunoglobulin-like receptor, two domains, long cytoplasmic tail, 2	3803	
	killer cell immunoglobulin-like receptor, two domains, short cytoplasmic tail, 2	100132285	
KIR2DS2;KIR2DL5A;KIR2DL2;KIR2DL3;KIR2DS4	killer cell immunoglobulin-like receptor, two domains, short cytoplasmic tail, 2	100132285	Hs04190776_gH
	killer cell immunoglobulin-like receptor, two domains, long cytoplasmic tail, 2	3803	
	killer cell immunoglobulin-like receptor, two domains, long cytoplasmic tail, 3	3804	
	killer cell immunoglobulin-like receptor, two domains, long cytoplasmic tail, 5A	57292	
	killer cell immunoglobulin-like receptor, two domains, short cytoplasmic tail, 4	3809	
KIR2DS2;KIR2DS1;KIR2DS3;KIR2DL1;KIR2DS4	killer cell immunoglobulin-like receptor, two domains, short cytoplasmic tail, 2	100132285	Hs04190778_mH
	killer cell immunoglobulin-like receptor, two domains, long cytoplasmic tail, 1	3802	
	killer cell immunoglobulin-like receptor, two domains, short cytoplasmic tail, 1	3806	
	killer cell immunoglobulin-like receptor, two domains, short cytoplasmic tail, 3	3808	
	killer cell immunoglobulin-like receptor, two domains, short cytoplasmic tail, 4	3809	
KIR2DS3;KIR2DS5	killer cell immunoglobulin-like receptor, two domains, short cytoplasmic tail, 3	3808	Hs04190781_mH
	killer cell immunoglobulin-like receptor, two domains, short cytoplasmic tail, 5	3810	
KIR2DS3;KIR2DL2;KIR2DS2;KIR2DS5;KIR2DL3	killer cell immunoglobulin-like receptor, two domains, short cytoplasmic tail, 3	3808	Hs04190782_gH
	killer cell immunoglobulin-like receptor, two domains, long cytoplasmic tail, 2	3803	
	killer cell immunoglobulin-like receptor, two domains, long cytoplasmic tail, 3	3804	
	killer cell immunoglobulin-like receptor, two domains, short cytoplasmic tail, 2	100132285	
	killer cell immunoglobulin-like receptor, two domains, short cytoplasmic tail, 5	3810	
KLRC2;KLRC3	killer cell lectin-like receptor subfamily C, member 2	3822	Hs04192492_gH
	killer cell lectin-like receptor subfamily C, member 3	3823	
TNFSF10	tumor necrosis factor (ligand) superfamily, member 10	8743	Hs00921974_m1
GZMK	granzyme K (granzyme 3; tryptase II)	3003	Hs00157878_m1
IL12RB1	interleukin 12 receptor, beta 1	3594	Hs00538167_m1
IL12RB2	interleukin 12 receptor, beta 2	3595	Hs01548202_m1
TLR8	toll-like receptor 8	51311	Hs00152972_m1
LAMP1	lysosomal-associated membrane protein 1	3916	Hs00174766_m1
NCR2	natural cytotoxicity triggering receptor 2	9436	Hs00183113_m1
ICOS	inducible T-cell co-stimulator	29851	Hs00359999_m1
PDCD1	programmed cell death 1	5133	Hs01550088_m1
TLR1	toll-like receptor 1	7096	Hs00413978_m1
CD33	CD33 molecule	945	Hs01076281_m1
CXCR1	chemokine (C-X-C motif) receptor 1	3577	Hs00174146_m1
TLR3	toll-like receptor 3	7098	Hs01551078_m1
IL10	interleukin 10	3586	Hs00961622_m1
TBX21	T-box 21	30009	Hs00203436_m1
CXCR3	chemokine (C-X-C motif) receptor 3	2833	Hs00171041_m1
KLRK1;KLRC4-KLRK1	killer cell lectin-like receptor subfamily K, member 1	22914	Hs00183683_m1
	KLRC4-KLRK1 readthrough	100528032	
IRF7	interferon regulatory factor 7	3665	Hs01014809_g1
KLRC1	killer cell lectin-like receptor subfamily C, member 1	3821	Hs00970273_g1
CCL3	chemokine (C-C motif) ligand 3	6348	Hs00234142_m1
LILRB2;LILRB1	leukocyte immunoglobulin-like receptor, subfamily B (with TM and ITIM domains), member 2	10288	Hs00601427_g1
	leukocyte immunoglobulin-like receptor, subfamily B (with TM and ITIM domains), member 1	10859	
CCR7	chemokine (C-C motif) receptor 7	1236	Hs01013469_m1
MKI67	antigen identified by monoclonal antibody Ki-67	4288	Hs01032443_m1
B3GAT1	beta-1,3-glucuronyltransferase 1 (glucuronosyltransferase P)	27087	Hs00218629_m1
BIRC3	baculoviral IAP repeat containing 3	330	Hs00154109_m1
CCL2	chemokine (C-C motif) ligand 2	6347	Hs00234140_m1
CCL4	chemokine (C-C motif) ligand 4	6351	Hs99999148_m1
CCL5	chemokine (C-C motif) ligand 5	6352	Hs00174575_m1
BCL2	B-cell CLL/lymphoma 2	596	Hs99999018_m1
CD244	CD244 molecule, natural killer cell receptor 2B4	51744	Hs00900271_m1
CD38	CD38 molecule	952	Hs01120071_m1
CD69	CD69 molecule	969	Hs00934033_m1
CLEC2B	C-type lectin domain family 2, member B	9976	Hs00192860_m1
CTSB	cathepsin B	1508	Hs00947433_m1
CXCR4	chemokine (C-X-C motif) receptor 4	7852	Hs00237052_m1
CXCR6	chemokine (C-X-C motif) receptor 6	10663	Hs00174843_m1
DPP4	dipeptidyl-peptidase 4	1803	Hs00175210_m1
ENTPD1	ectonucleoside triphosphate diphosphohydrolase 1	953	Hs00969559_m1
FASLG	Fas ligand (TNF superfamily, member 6)	356	Hs00181225_m1
CD160	CD160 molecule	11126	Hs00199894_m1
GAPDH	glyceraldehyde-3-phosphate dehydrogenase	2597	Hs99999905_m1
GNLY	granulysin	10578	Hs00246266_m1
GZMB	granzyme B (granzyme 2, cytotoxic T-lymphocyte-associated serine esterase 1)	3002	Hs01554355_m1
HLADRA	major histocompatibility complex, class II, DR alpha	3122	Hs00219575_m1
IL16	interleukin 16	3603	Hs00189606_m1
IL21R	interleukin 21 receptor	50615	Hs00222310_m1
IL2Ra	interleukin 2 receptor, alpha	3559	Hs00907777_m1
IL6ST	interleukin 6 signal transducer (gp130, oncostatin M receptor)	3572	Hs00174360_m1
KLRD1	killer cell lectin-like receptor subfamily D, member 1	3824	Hs00233844_m1
LGALS9	lectin, galactoside-binding, soluble, 9	3965	Hs00371321_m1
KLRF1	killer cell lectin-like receptor subfamily F, member 1	51348	Hs00212979_m1
NCR3	natural cytotoxicity triggering receptor 3	259197	Hs00394809_m1
POLR2A	polymerase (RNA) II (DNA directed) polypeptide A, 220kDa	5430	Hs00172187_m1
PRDM1	PR domain containing 1, with ZNF domain	639	Hs00153357_m1
PRF1	perforin 1 (pore forming protein)	5551	Hs00169473_m1
PSMG2	proteasome (prosome, macropain) assembly chaperone 2	56984	Hs00220315_m1
SLAMF5	CD84 molecule	8832	Hs01547121_m1
SLAMF7	SLAM family member 7	57823	Hs00221793_m1
SMAD4	SMAD family member 4	4089	Hs00232068_m1
SPATA2	spermatogenesis associated 2	9825	Hs00195835_m1
TGFB1	transforming growth factor, beta 1	7040	Hs00998133_m1
NKG7	natural killer cell group 7 sequence	4818	Hs01120688_g1
TGFBR1	transforming growth factor, beta receptor 1	7046	Hs00610318_m1
TIA1	TIA1 cytotoxic granule-associated RNA binding protein	7072	Hs00254561_m1
TNF	tumor necrosis factor	7124	Hs00174128_m1
TNFAIP3	tumor necrosis factor, alpha-induced protein 3	7128	Hs00234713_m1
TNFRSF9	tumor necrosis factor receptor superfamily, member 9	3604	Hs00155512_m1
TNFSF13B	tumor necrosis factor (ligand) superfamily, member 13b	10673	Hs00198106_m1

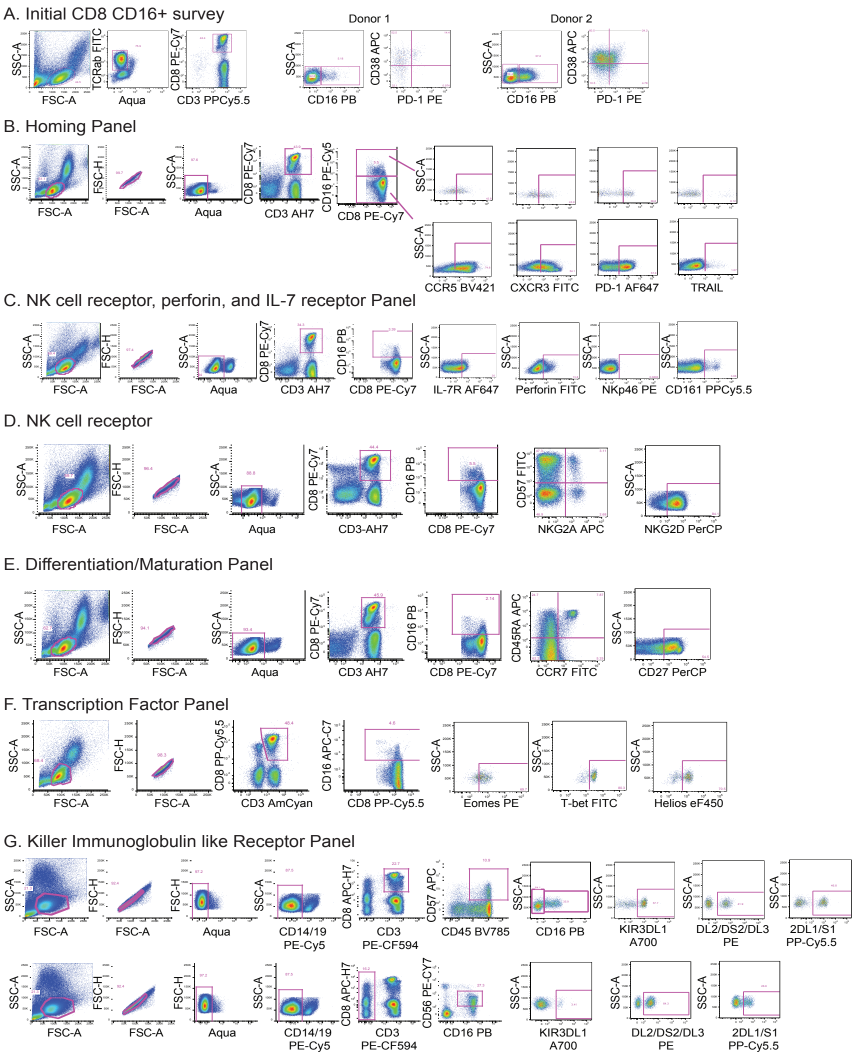

Supplemental Figure 1. Flow cytometry panels. Successive gating strategy used to characterize subpopulations of FcγRIIIa-expressing cell subsets using 7 flow cytometry panels. Gating strategies began by first identifying small lymphocytes through forward and side scatter profiles, single cells, and dead cell exclusion using an amine reactive dye. An initial survey identified an expanded FcγRIIIa+CD3+CD8+ cell subset within HIV+ donors, with differential PD-1 expression, as represented here **(A)**. Five additional flow cytometry panels were designed to further characterize this subset. Homing markers were characterized in CD16 positive *versus* negative CD8 T cells, with CCR5, CXCR3, PD-1 and TRAIL gated individually within each CD16 expression subset **(B)**. Additionally, CD16+ CD8 T cells were screened for surface and intracellular NK cell, innate-like markers through individual gating of IL-7R, Perforin, NKp46, and CD161 **(C)**, single gating on NKG2D, and all combinations of NKG2A and CD57, individually or together **(D)**. CD16+ CD8 T cells were also characterized for their differentiation and maturation markers through single gating on CD27, or all combinations of CD45RA and CCR7 **(E)**. Transcription factors T-bet, Eomes, and Helios were characterized alone or in combination within CD16+ CD8 T cells, CD16- CD8 T cells, as well as $CD56^{dim}$ FcγRIIIa+ NK cells **(F)**. Finally, surface KIR3DL1, KIRDL2/DS2/DL3, and KIR2DL1/S1 expression was measured in FcRγIIIa+ CD8 T cells and NK cells. CD8 T cells were gated on as CD3+CD8+ T cells, enriched for late-stage effector phenotype by gating on CD57+CD45+ cells, and further gated as CD16+. NK cells were identified by their CD3 negativity, followed by gating on $CD56^{dim}$CD16+ cells. All KIR gates were drawn as individual gates **(G)**.

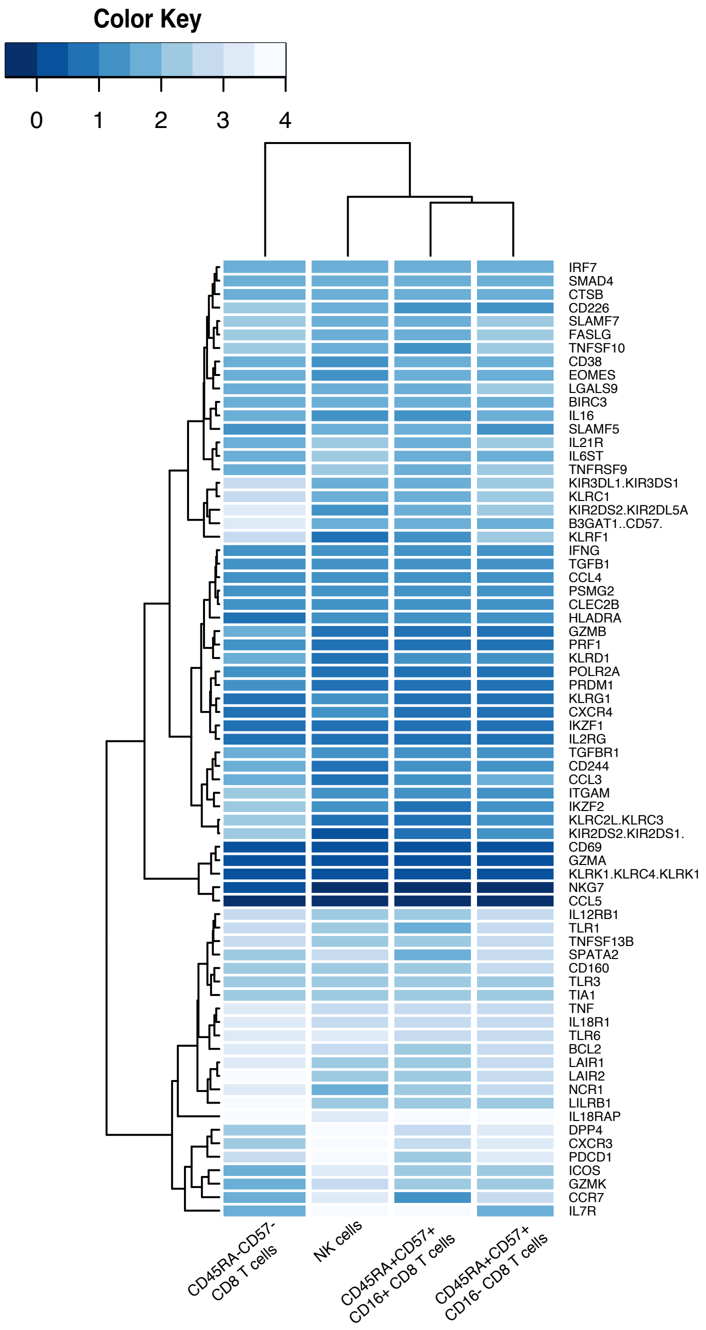

Supplemental Figure 2. Hierarchecal clustering of gene expression in sorted cell subsets. Two-way hierarchical clustering of gene expression in sorted subsets of CD8 T cells; CD45RA-CD57-, CD45RA+CD57+FcγRIIIA-, CD45RA+CD57+FcγRIIIA+, as well as CD56dim FcγRIIIA+ NK cells. Data is normalized 2^ΔCt values.
